# Asymmetric Introgression in the Horticultural Living Fossil *Cycas* Sect. *Asiorientales* Using a Genome-Wide Scanning Approach

**DOI:** 10.3390/ijms14048228

**Published:** 2013-04-15

**Authors:** Yu-Chung Chiang, Bing-Hong Huang, Chun-Wen Chang, Yu-Ting Wan, Shih-Jie Lai, Shong Huang, Pei-Chun Liao

**Affiliations:** 1 Department of Biological Sciences, National Sun Yat-sen University, Kaohsiung 80424, Taiwan; E-Mail: yuchung@mail.nsysu.edu.tw; 2 Department of Biological Science and Technology, National Pingtung University of Science and Technology, Pingtung 91201, Taiwan; E-Mails: sohjiro4321@yahoo.com.tw (B.-H.H.); myet556610@gmail.com (Y.-T.W.); s6163628@yahoo.com.tw (S.-J.L.); 3 Department of Life Science, National Taiwan Normal University, Taipei 116, Taiwan; E-Mail: chunwen@tfri.gov.tw (C.-W.C); biofv057@ntnu.edu.tw (S.H.); 4 Taiwan Forestry Research Institute, Technical Service Division, Taipei 10066, Taiwan

**Keywords:** introgression, AFLP, *Cycas revoluta*, *Cycas taitungensis*, horticulture

## Abstract

The Asian cycads are mostly allopatric, distributed in small population sizes. Hybridization between allopatric species provides clues in determining the mechanism of species divergence. Horticultural introduction provides the chance of interspecific gene flow between allopatric species. Two allopatrically eastern Asian *Cycas* sect. *Asiorientales* species, *C. revoluta* and *C. taitungensis*, which are widely distributed in Ryukyus and Fujian Province and endemic to Taiwan, respectively, were planted in eastern Taiwan for horticultural reason. Higher degrees of genetic admixture in cultivated samples than wild populations in both cycad species were detected based on multilocus scans by neutral AFLP markers. Furthermore, bidirectional but asymmetric introgression by horticultural introduction of *C. revoluta* is evidenced by the reanalyses of species associated loci, which are assumed to be diverged after species divergence. Partial loci introgressed from native cycad to the invaders were also detected at the loci of strong species association. Consistent results tested by all neutral loci, and the species-associated loci, specify the recent introgression from the paradox of sharing of ancestral polymorphisms. Phenomenon of introgression of cultivated cycads implies niche conservation among two geographic-isolated cycads, even though the habitats of the extant wild populations of two species are distinct.

## 1. Introduction

Introgression usually happens in contact zones of sympatric or parapatric species even they have diverged for millions of years after speciation [1]. In contrast to the sympatry or parapatry, introgression between allopatrically distributed species is more equivocal as the longer isolation results in deep divergence and lower chance for introgression, but species of recent allopatry have higher opportunity for introgression than species of sympatry in nature [2]. In addition, introgression occurs more easily in species of niche conservation than species of niche specialization when they are secondarily contacting [3]. Horticulturally, the introduction of alien species could accelerate the opportunity of interspecific gene flow, break the reproductive isolation, even create new chimera by hybridization between deep diverged species [4,5]. Therefore, degrees of introgression between horticulturally introduced species and native species provide clues for exploring the mechanism of species divergence in different geographic habitats.

Cycads are the classic living fossils with long evolutionary history but recently and rapidly diversified since the late Miocene [6]. Geographical isolation and small effective population sizes might be the reason for the rapid diversification [6]. The eastern Asian *Cycas* section *Asiorientales* is composed of two allopatric species *C. revoluta* and *C. taitungensis*, distributed widely in the Ryukyu archipelagos and Fujian Province of China and endemic to Taiwan, respectively. These two eastern Asian cycad species could coalesce to approximate 350 million years ago (mya) as inferred from fluctuations in very recent demography since around 5~3.5 mya [7]. The paraphyletic relationship was also suggested as a consequence of historical demographic fluctuations with past gene flow among ancestral populations [7].

*Cycas revoluta* is distributed around islands of the southern Japan and the eastern China, and widely planted in eastern Asia for horticultural reason. In contrast to the wider distributed and planted *C. revoluta*, *C. taitungensis* is endemic and restrictedly distributed in eastern Taiwan. Plantation of *C. taitungensis* is also found locally in the eastern Taiwan. Earlier unlawful lumbering and habitat destruction limited the population expansion of the native populations of *C. taitungensis* in Taiwan. Invasion of *Aulacaspis* spp. due to the introduction of foreign cycads in recent years severely infects the native *C. taitungensis* and results in high mortality in wild populations. Even though there is an endangered situation of extant populations, the genetic diversity of *C. taitungensis* does not appear low as estimated by either plastid and ribosomal DNA [7,8] or isozymes [9]. Low, even insignificant, genetic differentiation between populations of *C. taitungensis* was also reported, which is unusual for the species constrained by migratory capability of pollinators and seed carriers [8]. Restricted distribution and low migration rates for pollination decreased the probability of interspecific gene flow. However, *C. taitungensis* and *C. revoluta* still share identical genotypes and revealed paraphyletic relationships, which was suggested as a consequence of sharing high degrees of common ancestral polymorphisms [7]. Although Chiang *et al.*[7] indicated that the interspecific gene flow is greatly restricted between the extant wild populations by geographic isolation and low dispersability, gene flow between sympatrically cultivated samples were not examined yet. Whether such ancestral genetic compatibility still persisted between extant species is still unknown.

Hybridization between *C. taitungensis* and phylogenetically distant *C. ferruginea* was also performed in the botanical garden for examining the maternal inherited plastid genome [10]. However, the natural hybridization between these geographical isolated species is not reported. Genetic introgression between related plant species is probably more frequently than what we thought [4] through the process of gene transfer between plastid and nuclear genomes [11]. Sympatric growing of *C. revoluta* and *C. taitungensis* in gardens could enhance interspecific pollen (microspore) flow, and the amount of compatible microspores dictates the rate of introgression [12]. Cycad is mostly considered as entomophily (insect-pollination) [13,14] while the anemophily (wind-pollination) in certain cycad species could be a newly derived trait, *i.e.*, autapomorphy [13]. *Cycas revoluta* is also entomophilic by Coleoptera insects [15]. However, the trait of anemophily is also observed in *C. revoluta* although the amount of airborne pollens drop quickly in male cones distant from >2 [15], which supports Chiang *et al.*’s [7] speculation of restricted gene flow but also implies higher probability of successful pollinating via either insects or wind between individuals growing nearby.

For horticultural reasons, *C. revoluta* is introduced and widely planted in gardens, schools, and as the shade trees in Taiwan, which means that *C. revoluta* is not as distant from its relative *C. taitungensis*. In considering pollination compatibility of interspecific *Cycas* species [10], a prediction of enhanced introgression between *C. revoluta* and *C. taitungensis* by horticultural introduction of *C. revoluta* was made. Examination of genetic composition of individuals both in the wild and in the gardens in Taiwan was performed to clarify the prediction of introgression by horticulture. We hypothesized that interspecific gene flow increases the shared genetic polymorphisms in the cultivated individuals but not in the allopatrically wild populations. Therefore, we compared the genetic composition of the wild and cultivated samples of both *C. revoluta* and *C. taitungensis* using multilocus markers (*i.e.*, the amplified fragment length polymorphisms, AFLPs) for clarifying the interspecific gene flow at the artificially sympatric areas (e.g., gardens).

Neutral genes that experienced different rates of gene flow provide hints for investigating the asymmetric gene flow [16]. For evaluating the genetic impact of the horticultural introduction of *C. revoluta* on the native *C. taitungensis*, population genetic analyses with the multilocus neutral loci were used to address two specific objectives of this study: (1) to reevaluate the genetic diversity of the cultivated and extant wild populations of *C. taitungensis* by multilocus marker and (2) to evaluate the degrees of introgression between *C. revoluta* and *C. taitungensis* in Taiwan. In this study, we also discuss the mechanisms (e.g., geographic isolation or niche specialization) of the divergence of these two phylogenetically related species through an examination of the horticultural introgression.

## 2. Results

### 2.1. Sampling and Neutrality Test of AFLP Polymorphisms

**Figure 1:**
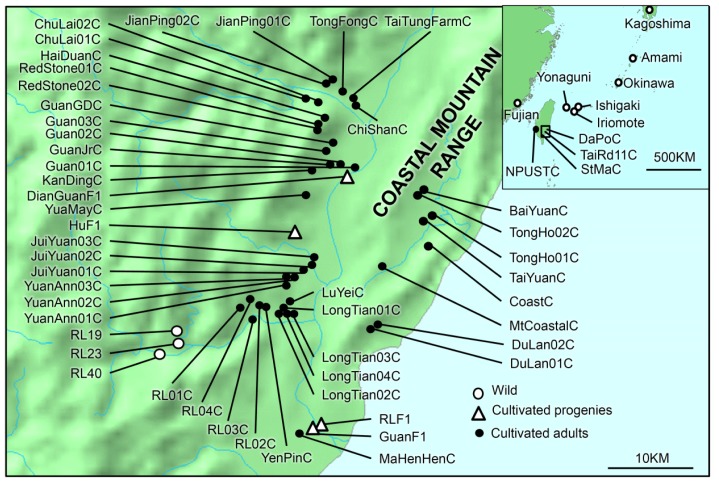
Map of sampling sites in this study. The large panel indicates the sampling sites of *C. taitungensis* in the southeast Taiwan and the upper-left panel indicates the sampling sites of *C. revoluta*. Wild populations, cultivated adults and progenies are marked in hollow circles, full circles, and hollow triangles, respectively. Codes of the sampling sites correspond to Table A1.

**Figure A1:**
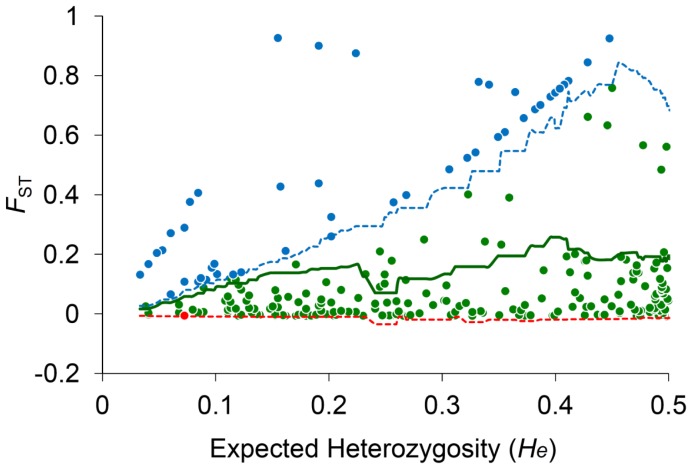
Examination of neutrality of AFLP loci based on Beaumont and Nichols’s [56] method by program Mcheza [46]. Loci located within the 95% confidence intervals are taken as neutral loci (solid dots) and the positive (open diamonds) and negative outliers (open squares) are taken as candidate loci under positive and balancing selection, respectively.

**Figure A2:**
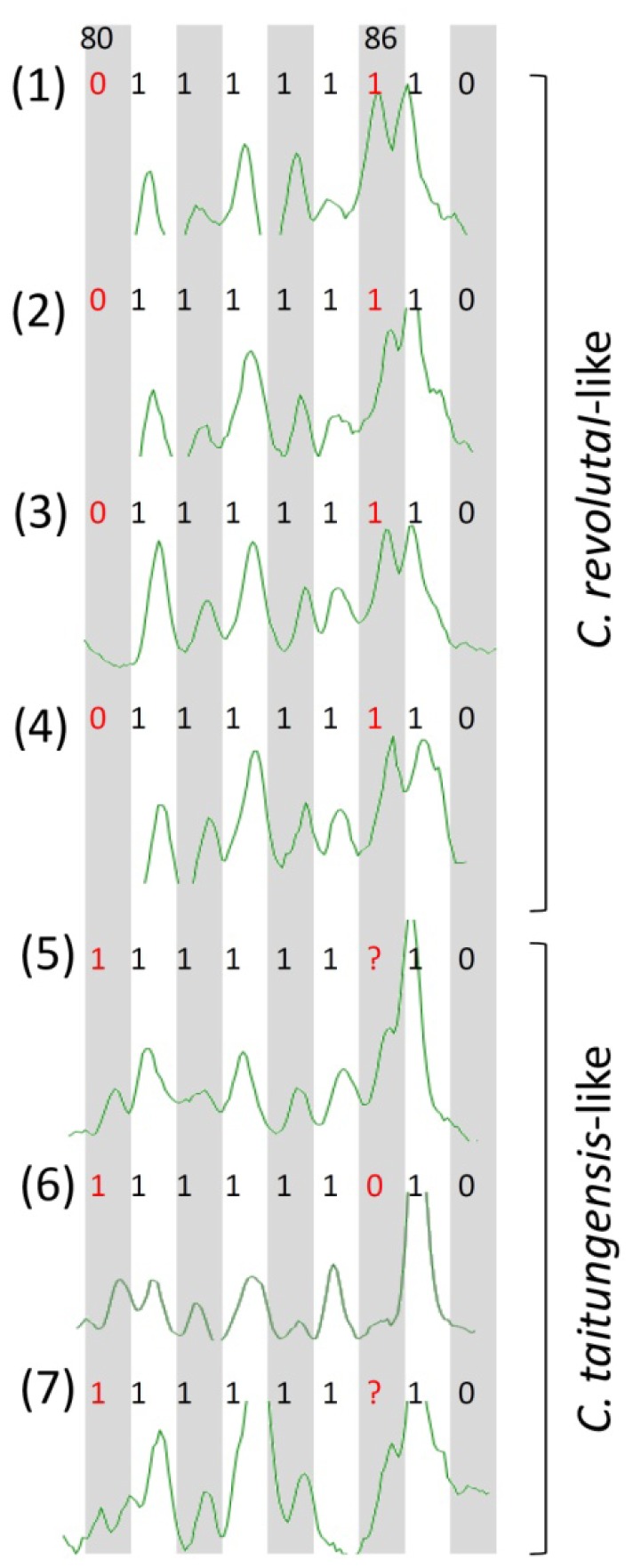
Examples of the ABI prism graphs revealing the loci with present (1) and absent (0) peaks. The question mark (?) indicates the missing (ambiguous) allele. Examples were separated to seven populations: (**1**) the wild population of *C. revoluta* in Ryukyus; (**2**) the wild population of *C. revoluta* in Fujian; (**3**) the *C. revoluta*-like cultivated population of *C. revoluta* in Taiwan; (**4**) the *C. revoluta*-like cultivated population of *C. taitungensis* in Taiwan; (**5**) the wild population of *C. taitungensis* in Taiwan; (**6**) the *C. taitungensis*-like cultivated population of *C. taitungensis* in Taiwan; and (**7**) the *C. taitungensis*-like cultivated population of *C. revoluta* in Taiwan. The polymorphic loci represented in this example graph are indicated in red (*i.e.*, the 80 and 86 sites), which separate these individuals to the *C. revoluta*-like and the *C. taitungensis*-like groups in genetic components.

Twenty one and 62 samples of *C. revoluta* and *C. taitungensis* were collected from wild populations, respectively, and 29 cultivated samples of *C. revoluta* and 151 and 134 cultivated adults and seedlings of *C. taitungensis* were collected in the South and Southeast Taiwan (Figure 1 and Table A1). In total, 311 polymorphic loci were obtained from 397 samples of both *C. revoluta* and *C. taitungensis*. One and 48 of the 311 loci had significantly lower and higher *F*_ST_ values deviating from 95% confidence intervals, respectively, and were defined as negative and positive outliers, respectively (Figure A1). The rest of the 262 loci located within the 95% confidence intervals were considered as neutral loci. Example panels of species- and population-specific loci detected by the AFLP genotyping are provided in Figure A2.

### 2.2. Genetic Diversity

Both *C. revoluta* and *C. taitungensis* have 77.86% polymorphic neutral loci (Table 1). *Cycas revoluta* revealed slightly higher genetic diversity than *C. taitungensis* in total samples in Shannon’s information index (*I* = 0.422 ± 0.017 and 0.401 ± 0.016, respectively, *p* = 0.0140, Student’s *t* test) and the expected heterozygosity (*h* = 0.286 ± 0.012 and 0.266 ± 0.011, respectively, *p* = 0.0294) but insignificantly different in indices number of effective alleles (*Ne* = 1.504 ± 0.024 and 1.446 ± 0.021, respectively, *p* = 0.1261) and unbiased heterozygosity (*uh* = 0.292 ± 0.012 and 0.267 ± 0.011, respectively, *p* = 0.1208) even though the sample size of *C. revoluta* is relatively small (Table 1). However, when only comparing the wild population samples between two species, *C. revoluta* has relatively smaller but insignificant genetic diversity than *C. taitungensis* in all indices (*p* = 0.9051, 0.3934, 0.5477 and 0.8968 in *Ne*, *I*, *h* and *uh*, respectively). The relatively lower genetic diversity of wild *C. revoluta* than *C. taitungensis* is consistence with estimates of plastid DNA sequences by Huang *et al.*[8] and Kyoda and Setoguchi [17] but inconsistent with Chiang *et al.*[7]. Although the different estimates could be due to sample sizes, the probability of heterogeneous evolutionary rates in different genomic markers cannot be excluded. When comparing with the cultivated adults, *C. taitungensis* has relatively higher genetic diversity than the introduced *C. revoluta* in *I* and *h* (*p* = 0.0195 and 0.0268, respectively) but insignificantly different in the other indices; non-significant differences of diversity indices (*p* > 0.05) were also estimated between cultivated adults and progenies of *C. taitungensis*, between cultivated progenies of *C. taitungensis* and cultivated adults of *C. revoluta*, and between wild populations and cultivated adults or progenies of both species (Table 1). Detailed genetic diversity of each population is shown in Table 1.

### 2.3. Species-Associated Loci

**Figure A3:**
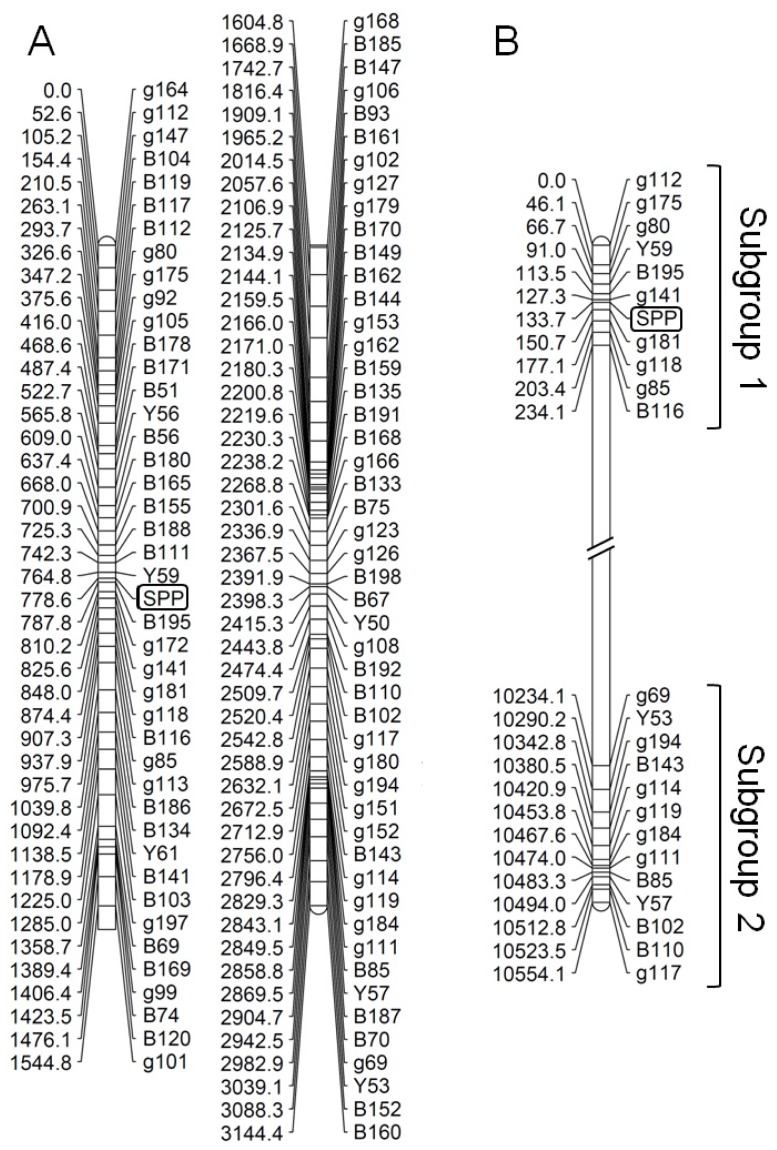
Linkage maps show the species-associated loci estimated by the maximum likelihood mapping algorithm using JoinMap ver. 4.0 [53]. Linkage group of species-associated loci estimated by criteria of (**A**) LOD 3.0 and (**B**) LOD 6.0 are shown. Two linkage subgroups at LOD 6.0 were inferred. The code SPP indicates the “character” as species.

Linkage group with the character “species”, named species-associated loci, was detected by neutral loci using loose (LOD 3.0) and strict criteria (LOD 6.0). These loci were chosen as the loci diverged after species divergence. Among 262 neutral loci, 91 and 23 loci were detected linked with the character “species” at linkage threshold LOD 3.0 and LOD 6.0, respectively (Figure A3). The abundance of species-associated loci at loose and strict criteria composes about one-third and one-tenth neutral loci. In addition, the linkage-group loci at LOD 6.0 revealed two linkage subgroups, named linkage subgroup 1 (tightly linked with “species”) and subgroup 2, composed of 10 and 13 loci, respectively (Figure A3B). Four sets of loci (262 neutral loci, 91 species-associated loci at LOD 3.0, 23 species-associated loci at LOD 6.0, and 10 species-associated loci of linkage subgroup 1 at LOD 6.0) were used for the further PCoA and Bayesian clustering analysis.

### 2.4. Principle Coordinate Analyses

**Figure 2:**
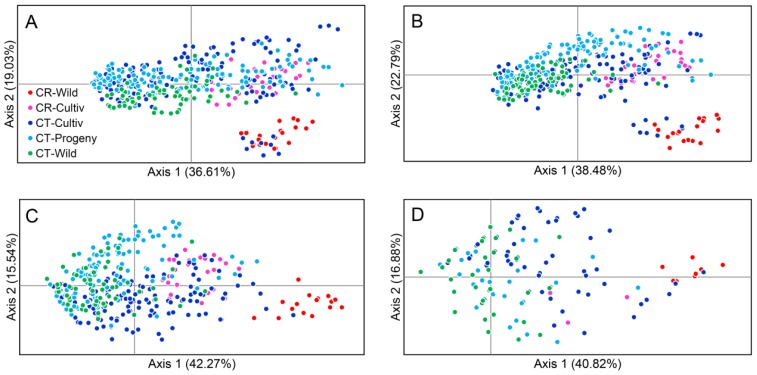
The first two axes plots of principle coordinate analysis (PCoA) calculated using (**A**) 262 neutral loci; (**B**) 91 species-associated loci determined under criteria LOD 3.0; (**C**) 23 species-associated loci determined under criteria LOD 6.0; and (**D**) 10 species-associated loci of the linkage subgroup 1 under criteria LOD 6.0. Abbreviations CR ad CT indicate *C. revoluta* and *C. taitungensis*, respectively; Wild, Cultiv, and Progeny indicate the wild populations, the cultivated adults, and the cultivated seedlings, respectively.

Genetic homogeneity of wild and cultivated samples between *C. revoluta* and *C. taitungensis* is tested by PCoA. In comparison of wild populations by 262 neutral loci, genetic compositions of two species cannot be distinguished at the first axis (explained 36.1% variations) but are obviously distinct by the first two axes (explained 55.64% variations) (Figure 2A). Similar result was also detected in species-associated loci at linkage threshold LOD 3.0 that the resolution of only first axis (38.48%) is worse but is better in the first two axes (61.27%) (Figure 2B), while genetic distinction is clear at the first axis at the stricter criterion LOD 6.0 (explains 42.27% variations) or its linkage subgroup 1 (explains 40.82% variations) (Figure 2C,D).

When comparing cultivated samples to the wild populations, genetic compositions of *C. revoluta* are indistinguishable at the first axis by 262 neutral loci or species-associated loci at LOD 3.0 but can be obviously distinguished at the first two axes (Figure 2A,B). However, obvious distinction is shown at the first axis under stricter criterion of species association (Figure 2C,D), which implies the shift of genetic components in cultivated samples of *C. revoluta* from the wild populations. In contrast to *C. revoluta*, both cultivated adults and progenies of *C. taitungensis* have wider ranges of genetic composition, covering the genetic distribution of wild populations of *C. taitungensis* and cultivated samples of *C. revoluta* in four loci sets (Figure 2). This result also indicates shifts of genetic components of partial samples of cultivated *C. taitungensis* from wild populations. Furthermore, a very clear pattern was found of the cultivated samples of *C. revoluta* grouped with the cultivated samples of *C. taitungensis*, especially with the cultivated adults, in all four loci-set analyses (Figure 2). However, the genetic distribution of the cultivated *C. revoluta* is not entirely covered by wild samples but by cultivated samples of *C. taitungensis*. It is also noticeable that certain samples of cultivated adults of *C. taitungensis* are grouped to the wild *C. revoluta*. We are not sure whether it implies a long-distant introgression from Ryukyus or Fujian, but these results clearly indicate more severely genetic admixture among cultivated cycads than among allopatrically wild populations.

### 2.5. Bayesian Clustering Analysis

**Figure 3:**
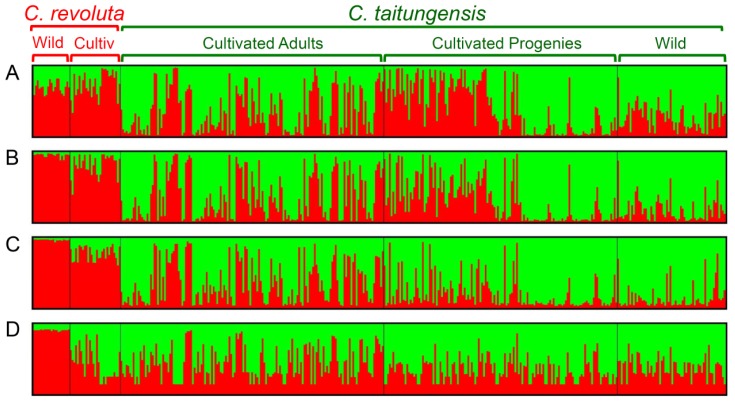
Assignment test by Bayesian clustering analysis of wild populations and cultivated samples of *C. revoluta* and *C. taitungensis* at clustering number *K* = 2. Patterns of genetic composition of each individual are detected by (**A**) 262 neutral loci; (**B**) species-associated loci at criteria LOD 3.0; and (**C**) LOD 6.0; and (**D**) the loci of linkage subgroup 1 of LOD 6.0.

**Figure 4:**
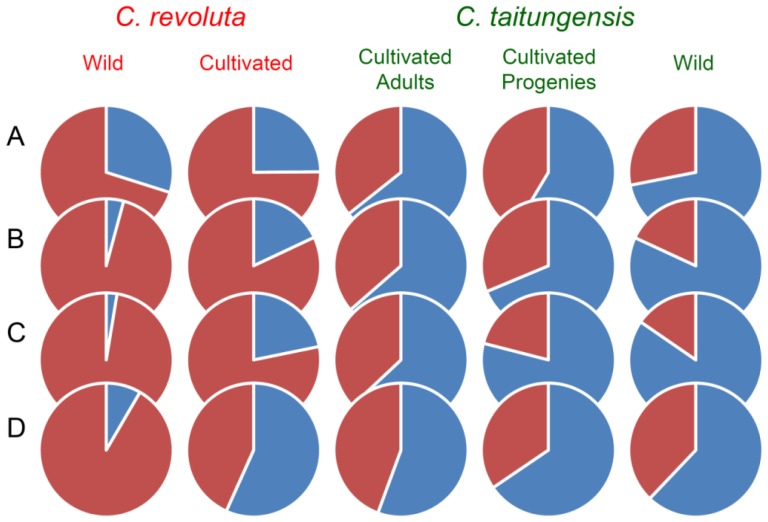
Average genetic composition of wild populations and cultivated samples of *C. revoluta* and *C. taitungensis*, detected by (**A**) 262 neutral loci; (**B**) species-associated loci at criteria LOD 3.0; and (**C**) LOD 6.0; and (**D**) the loci of linkage subgroup 1 of LOD 6.0.

**Figure A4:**
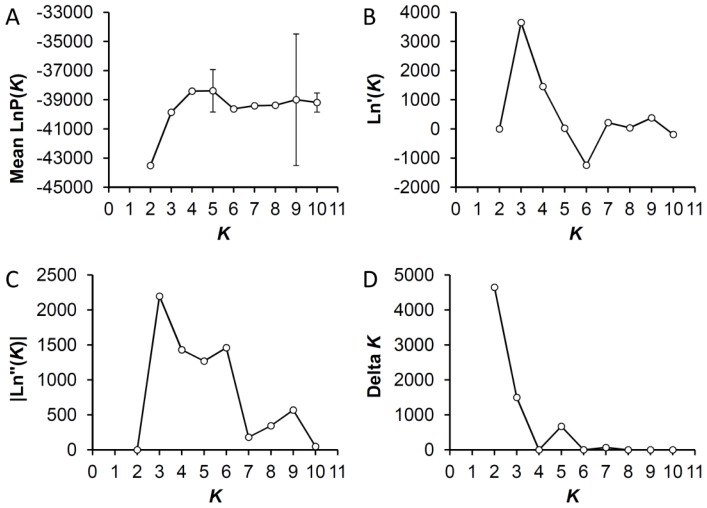
Evaluation of the best grouping number (*K*) of the Bayesian clustering analysis using Evanno *et al.*’s [51] methods. (**A**) Mean L_n_P(*K*); (**B**) L_n_′(*K*), equation to L_n_′(*K*) = L_n_P(*K*) − L_n_P(*K* − 1); (**C**) |L_n_′′(*K*)|, equation to |L_n_′(*K* + 1) − L_n_′(*K*)|; and (**D**) Δ*K*, equation to *m*(|*L*′′(*K*)|)/*s*[*L*(*K*)], where *m* and *s* are the mean and standard deviation, respectively.

In the Bayesian clustering analysis, the best group manner is inferred as two by the Δ*K* evaluation (Δ*K* = 1154.354 when *K* = 2) when using the 262 neutral loci (Figure A4). However, the grouping pattern is not completely consistent with taxonomic grouping, *i.e.*, revealed an admixture genetic composition, especially for the cultivated samples of *C. taitungensis* (Figure 3A). Also, for resolving the paradox of sharing common ancestral polymorphisms or recent introgression, the Bayesian clustering analysis was redone using species-associated loci. The Bayesian clustering analysis still revealed mosaic genetic composition at the species-associated loci at both criteria of species association (*i.e.*, LOD 3.0 and LOD 6.0, Figure 3B,C, respectively). Cultivated samples of both species were apparently composed of higher frequencies of alien genes than the wild populations (Figure 4A–C). However, the degree of genetic mosaicism increases when using the loci of linkage subgroup 1 of LOD 6.0 (Figure 3D), and even cultivated samples of *C. revoluta* were inferred to be composed of relatively higher genetic components belong to *C. taitungensis* than belong to *C. revoluta* itself (Figure 4D). This implied that (1) these ten loci of linkage subgroup 1 of LOD 6.0 could be locally selected or adapted in Taiwan, or (2) these ten loci revealed an opposite direction of introgression from *C. taitungensis* to *C. revoluta.* Because the outlier-loci have been eliminated in clustering analysis, the possibility of local adaptation can be excluded, and the introgression from *C. taitungensis* to *C. revoluta* is more appropriate to explain the high genetic components of *C. taitungensis* in cultivated *C. revoluta* (Figures 3D and 4D).

## 3. Discussion

Cycad is commonly planted for horticultural reasons for a long time. Introduction history of *C. revoluta* into Taiwan is probably decades or hundreds years ago, since the Chinese Hans or Japanese colonization. Horticultural introduction spreads *C. revoluta* in Asia and leads this species to secondarily contact with other cycads (e.g., *C. taitungensis* in Taiwan) since they diverged. Our genomic survey by AFLP multilocus scans evidenced that sympatric plantation increases the opportunity of introgression. This study evidences asymmetric introgression among invading and native cycads and suggests their niche conservatism after speciation by geographic isolation.

### 3.1. Asymmetric Introgression between Cycad Species in Taiwan

Successful pollination of *C. revoluta* is limited by distance [15]. Allopatric distribution of wild populations of *C. revoluta* and *C. taitungensis* restricts the pollen flow from each other, while the horticultural introduction increases the chance of interspecific gene flow. Introgression could be considered as a kind of genetic invasion [1,16], and direction of introgression is commonly considered from native species into the invaders due to population size effect [16,18]. However, the contrast phenomenon of introgression from invaders into natives is also reported in poplar [19], rice [20], and bitter melons of Taiwan [21]. In this case of *Cycas* in Taiwan, severe introgression from invaders (*C. revoluta*) to natives (*C. taitungensis*) is obviously revealed in the undistinguishable patterns of PCoA at the first axis (Figure 2) and revealed in the Bayesian clustering analysis (Figures 3 and 4), while the opposite-directional introgression is also detected, despite being relatively small (Figure 4). Based on the distribution pattern (wider distribution in *C. revoluta vs.* restricted distribution in *C. taitungensis*) and the paraphyletic relationship [7], *C. taitungensis* could be just a unique lineage of the ancestor of *C. revoluta* with more autapomorphies. Therefore, *C. revoluta* could be more incompatible to receive the alien genes from *C. taitungensis* while *C. taitungensis* is more compatible to receive plesiomorphic alleles from *C. revoluta*.

### 3.2. Paradox of Sharing Ancestral Polymorphisms and Recent Introgression

The shared polymorphisms are usually questioned as a consequence of common ancestral polymorphisms instead of introgression. For resolving this question, we redid the PCoA and Bayesian clustering analysis by the “species-associated loci”. If there was no introgression, the species-associated loci would differentiate well without admixture; in contrast, if introgression happens, the species-associated loci would represent admixture pattern. This assumption is similar to the concept of “divergence hitchhiking” [22] but we only considered the neutral loci rather than the “outlier loci” for eliminating the interference of adaptation or speciation genes. The wild populations that allopatrically distributed were used as template for determining the species-associated loci in order to detect the introgression between horticultural *C. revoluta* and *C. taitungensis*. Frequencies of the sharing polymorphisms apparently decreased in wild populations of both species by the reanalysis of Bayesian clustering analysis, but unvaried in cultivated samples (Figure 4B,C, in comparison of Figure 4A). This result indicated that the introgression occurred in cultivated cycads and evidenced the acceleration of introgression by horticultural introduction of *C. revoluta*.

### 3.3. Genetic Chimera of Cultivated Cycads

Obvious patterns that a broader and continuous genetic distribution of cultivated *C. taitungensis* covers the wild populations and cultivated *C. revoluta* while the grouping of cultivated *C. revoluta* is distinct from wild populations of both species are shown in PCoA (Figure 2). This is probably because the cultivated samples are composed of genes of both species by hybridizing recombination, *i.e.*, chimeric DNA [23,24]. It also implied that the introgression could only happen between cultivated samples but not between wild populations. However, the cultivated chimeric *C. taitungensis* could probably backcross with wild populations in short geographic distance, which explains (1) continuous and broader genetic distribution of cultivated *C. taitungensis* covering with the wild populations in PCoA (Figure 2) and (2) highly genetic admixture of wild samples of *C. taitungensis* in Bayesian clustering analysis (Figure 3). In contrast, both PCoA and Bayesian clustering analysis showed that the backcross of cultivated samples with long-distant wild populations seems rarer in *C. revoluta*, which is probably because of low dispersability of pollens [15] and seeds [7]. Genetic chimera explain not only the sustention of genetic diversity of horticultural *C. taitungensis* but also the distinction between cultivated and wild samples of *C. revoluta* (Table 1), which is broadly evidenced in microbes [25,26]. Hybridizing recombination would also raise the number of rare alleles [27,28] especially in those newly derived hybrids [29]. However, this speculation of genetic chimera is difficult to test by AFLP markers and we hope to test this further by codominant-marker surveys (e.g., by microsatellite DNAs) like the beautiful case of grapevine [30,31].

### 3.4. Niche Conservation Accelerates Sympatric Introgression

The two cycad species *C. revoluta* and *C. taitungensis* were geographically isolated with different habitats: *C. revoluta* mostly grows along coasts and is subjected to salt spray in Ryukyus and Fujian Province of China while *C. taitungensis* grows under forests along river valleys in Taiwan Island [7]. Therefore, what mechanism, *i.e.*, geographic isolation or adaptive divergence, results in the species divergence is curious. Since we know that degrees of gene flow decrease between organisms “if adaptation to a particular habitat determines where organisms mate [32]” but would recover between organisms of niche conservatism [33], estimating the interspecific gene flow could be useful for determining the mechanism of geographically or adaptively reproductive isolation. In other words, the interspecific gene flow might be recovered when organisms met (*i.e.*, secondary contact) through niche conservation; in contrast, if species divergence with niche specialization, the reproductive isolation would be retained by the incompatibility of adapted genes between species [34] or by eliminating immigrant alleles [35]. In this case, higher genetic admixture was shown in cultivated samples of two species than the allopatrically distributed wild populations, implying that the species divergence could be mainly affected by geographic isolation rather than adaptive divergence.

Although these two *Cycas* species grow in different environments in the wild, the growing condition of both species is similar, implying their broad adaptability without niche specialization. In addition, multiple extant Asian cycad species, including *C. taitungensis*, are allopatric and restrictedly distributed with small population sizes [36–40] and is considered as relicts from glacial refugia [8]. The small population size and geographic isolation from other populations increase the effect of genetic drift resulting in species divergence. However, the time to geographic isolation seemed not enough to complete the reproductive isolation and was broken off by transplantation. In fact, frequent hybridization could be seen in botanical gardens in several species whether naturally or artificially [29,41]. Artificial hybridization between *C. revoluta* and *C. taitungensis* is also successfully done by horticulturists [42,43]. Hybridization between *C. taitungensis* and *C. ferruginea* (sect. *Stangerioides*) was even performed in the botanical garden [10]. This indicates that the introgression could occur more easily among these living fossil cycads than what we thought when secondarily contacting.

Although the introgression between the *C. revoluta* and *C. taitungensis* has been proved by genetic analyses, morphological characters (leaf traits) that are commonly used to identify these two cycads do not change. The unchanged leaf traits of the cultivated *C. taitungensis*, such as the flat leaves and plane leaflet margins (in contrast to the deep keeled leaves and revolute leaflet margins of *C. revoluta*), reflect the fact of none or rare effects on the leaf character shift after introgression. The unchanged morphotypes of the genetically chimeric individuals have made the introgression an unseen threat to the native cycads.

## 4. Experimental Section

### 4.1. Sampling

The sampling of cycad species included two parts: the wild samples and the cultivated samples. In the sampling of wild populations, because the main purpose of this study focused on the genetic introgression of horticultural cycads in Taiwan, an indicative sampling of three individuals from each wild population of *C. revoluta* were performed; in contrast, *C. taitungensis* is only restricted distributed in the Hong-Yeh valley (the preserve areas of 19th, 23rd and 40th Compartment of Yen-Ping Area, Taitung County) and sparse in the Coastal Mountain Range of the southeastern Taiwan, the sampling of wild *C. taitungensis* only focused on the main wild population at three compartments of Hong-Yeh valley (Figure 1). In the part of cultivated sampling, the sampling areas were concentrated on sympatrically distributed areas of two cultivated cycad species in the southern and southeastern Taiwan. Species identification of horticultural samples was based on two distinguished leaf characters: flatter leaves and plane leaflet margins in *C. taitungensis vs.* deep keeled leaves and revolute leaflet margins in *C. revoluta*. In addition to the adults, the seedlings (progenies) of *C. taitungensis* in nursery gardens were also collected. In total, 397 individuals were collected for genetic analyses. Detailed information of the sampling sites is listed in Table A1.

### 4.2. DNA Extractions and AFLP Genotyping

Total genomic DNA was extracted with cetyl trimethylammonium bromide (CTAB) method [44]. The AFLP was performed following the method developed by Vos *et al.*[45] with little modification. Two restricted enzymes *Eco*RI (10 Unit) and *Mse*I (10 Unit) (New England Biolabs, Beverly, MA, USA) were used to digest the sample DNA with the following amplification by the pre-selected primers Eco+A (GACTGCGTACCAATTCA) and Mse+C (GATGAGTCCTGAGTAAC) and the selected primer pairs Eco+AGT/Mse+CTA, Eco+ACG/Mse+CTC, and Eco+AAT/Mse+CTG. These primers were labeled with florescence dye (6FAM, JOE, and TAMRA, respectfully), and the genotyping was performed on ABI Prism 3730XL (Applied Biosystems, Foster City, CA, USA). LIZ600 was used as size standard and peak size detection was conducted by Peak Scanner ver. 1.0 (Applied Biosystems, Foster City, CA, USA). Detailed methods are provided in Supplementary Materials.

### 4.3. Data Scoring and Data Analyses

The present and absent loci of the AFLP bands (peaks) ranged from 50 to 300 bps were scored as 1 and 0, respectively. For evaluation of neutrality of AFLP loci, the Dfdist approach was used by the program McHeza [46]. A strict criterion of 95% confidence interval (CI) was set for defining the neutral-evolving loci. The percentage of polymorphic loci (PPL), number of effective alleles (*Ne*), expected heterozygosity (*h*), unbiased heterozygosity (*He*), and Shannon’s information index (*I*) were estimated using the neutral loci by GenAlEx ver. 6.3 [47] in order to reveal the genetic diversity of wild and cultivated populations of *C. taitungensis* and *C. revoluta*. The principle coordinate analysis (PCoA) and the model based Bayesian clustering analysis were performed to evaluate the degrees of genetic admixture and genetic structure. The PCoA and the Bayesian clustering analysis were conducted by GenAlEx ver. 6.3 [47] and STRUCTURE ver. 2.3.3 [48–50], respectively. Simulation results of the best grouping number *K* analyzed by STRUCTURE were evaluated using Δ*K*[51] by STRUCTURE HARVESTER ver. 0.6.8 [52] (see Figure A4). Detailed descriptions and program settings were available in Supplementary Materials.

In order to ascertain the paradox of sharing common ancestral polymorphism [7] from introgression, we used the concept of linkage to determine the “species-associated” loci by detecting the samples from allopatrically wild populations of *C. revoluta* and *C. taitungensis*. This hypothesis was made under the premise of the species-associated loci were diverged after species divergence. Therefore, the divergence of species-associated loci would follow the divergence of species and would “link” with the character “species”. Wild populations of allopatric distribution were used for looking for the species-associated loci. The “species” was treated as one character to join the 262 neutral loci to determine the linkage group using JoinMap ver. 4.0 [53]. Low and high linkage thresholds were set at the LOD 3.0 and LOD 6.0 to evaluate the loci that associated with “species”, respectively. The other para followed the default setting of JoinMap. After determining the species-associated loci, the PCoA and Bayesian clustering analysis were redone for all samples (including the wild populations and cultivated samples) for detecting whether the introgression was occurred after horticultural introduction.

## 5. Conclusions

Introgression between sympatrically cultivated *C. revoluta* and *C. taitungensis* reveals incomplete reproductive isolation between deep divergent species of cycads. The natural introgression among horticultural individuals from different sources also supports the inference that the selection is not necessary for introgression [16]. Genetic evaluation of the wild populations of both *Cycas* species indicates a more severe impact on population genetic structure of the native *C. taitungensis* than *C. revoluta*. Detection of the divergence pattern of the species-associated loci helps to distinguish the sources of genetic admixture between the recent introgression and sharing common ancestral polymorphisms. Furthermore, asymmetric introgression is probably due to the demographic imbalance of these two species at the wave front for surfing [7,16], which could threat the native species by rapid spread of invasive genes [54,55]. The introgression hence becomes another important conservation issue of cycads beyond the illegal logging, habitat destruction, and the plague of vermin.

## Figures and Tables

**Table 1:** Genetic diversity of wild populations and cultivated samples of *C. revoluta* and *C. taitungensis* estimated using 262 neutral AFLP loci.

Species/Population	*N*	*Ne*	*I*	*h*	*uh*	%*P*
*Cycas revoluta*	50	1.504 ± 0.024	0.422 ± 0.017	0.286 ± 0.012	0.292 ± 0.012	77.86%
Wild population	21	1.406 ± 0.024	0.353 ± 0.017	0.236 ± 0.012	0.248 ± 0.013	69.47%
Cultivated-Adults	29	1.390 ± 0.024	0.343 ± 0.017	0.227 ± 0.012	0.235 ± 0.013	69.47%
*Cycas taitungensis*	347	1.446 ± 0.021	0.401 ± 0.016	0.266 ± 0.011	0.267 ± 0.011	77.86%
Wild population	62	1.410 ± 0.022	0.373 ± 0.016	0.246 ± 0.011	0.250 ± 0.012	77.10%
Cultivated-Adults	151	1.446 ± 0.022	0.397 ± 0.016	0.264 ± 0.011	0.266 ± 0.011	76.72%
Cultivated-Progeny	134	1.410 ± 0.021	0.374 ± 0.016	0.246 ± 0.011	0.248 ± 0.011	75.95%

*N*, sample size; *Ne*, number of effective alleles; *I*, Shannon’s Information Index; *h*, expected heterozygosity; *uh*, unbiased expected heterozygosity; %*P*, percentage of polymorphic loci.

## Appendix

### Methods

#### A1.1. Methods for DNA Extractions and AFLP Genotyping

Fresh leaves were dried by silica gel immediately and ground to powder by liquid nitrogen after being carryied to the laboratory. Total genomic DNA was extracted by the cetyl trimethylammonium bromide (CTAB) method [44]. The extracted DNA was dissolved in 1X TE buffer and stored at −20 °C.

For the multilocus genome-scan approach, we adopted the amplified fragment length polymorphism (AFLP) for genetic assessment. The AFLP was performed following the method developed by Vos *et al.*[45] with little modification. A total of 250 ng DNA were digested by restriction enzymes, EcoRI (10 Unit) and MseI (10 Unit) (New England Biolabs, Beverly, MA, USA); in total 25 μL reaction in 37 °C for 3 h, followed by 70 °C for 15 min to inactivate enzyme activity. The 5 μL digested products were added to 15 μL ligation mix with 5 pmol EcoRI adapter, 50 pmol MseI adapter, and 1 Unit T4 DNA ligase (New England Biolabs, Beverly, MA, USA) in 16 °C for 1 h then 37 °C for 3 h. Ligated products were pre-selected by 0.5 mM primers Eco+A (GACTGCGTACCAATTCA) and Mse+C (GATGAGTCCTGAGTAAC), with 2.5 nmole dNTP, 3 nmole MgCl2, 0.2 μL 1% BSA and 1 Unit DNA Taq polymerase. Amplification reactions were performed under 94 °C for 30 s, 56 °C for 1 min, and 72 °C for 1 min with 20 cycles. Pre-selected products were used as template for selective amplification. Selective amplifications were conducted by using 0.5 mM primer pairs Eco+AGT/Mse+CTA, Eco+ACG/Mse+CTC, and Eco+AAT/Mse+CTG. These primers were labeled with florescence dye (6FAM, JOE, and TAMRA, respectfully), with 3 nmole MgCl2, 2.5 nmole dNTP, and 1 Unit DNA Taq polymerase. Selective PCR was set for 94°C for 2 minutes for reaction activation, followed by a total of 25 cycles of 94 °C for 30 s, 65 °C for 30 s (decreasing 1 °C every cycle until it reached 56 °C) and 72 °C for 1 min, with subsequently 72 °C for 30 min for final extension. Concentration of selective amplified products was checked under 1.5% agarose gels. Genotyping was performed on ABI Prism 3730XL (Applied Biosystems, Foster City, CA, USA). LIZ600 was used as size standard and peak size detection was conducted by Peak Scanner ver. 1.0 (Applied Biosystems, Foster City, CA, USA).

#### A1.2. Data Scoring, Genetic Diversity, and Population Structure

AFLP bands with the same migration distances were considered homologous loci and scored manually as present (1) or absent (0). The sizes of the AFLP bands scored ranged from 50 to 300 bps. For evaluation of neutrality of AFLP loci, the Dfdist approach which evaluates the distribution of heterozygosity and genetic differentiation (*F*_ST_) of each locus [56,57] was used by the program McHeza [46]. Since we wanted to eliminate the interference of adaptive effect for evaluating introgression, a strict criterion of 95% confidence interval (CI) rather than the 99% CI was set for defining the neutral-evolving loci. Two strategies were used for detecting the loci with outlier *F*_ST_: (1) the mean *F*_ST_ was calculated by McHeza and forced the simulations according to the mean *F*_ST_; (2) we ran McHeza as in Strategy 1 but ran the simulation after removing the loci outside the 95% CI, as recommended by Antao *et al.*[58]. One million Markov chain simulations were performed. Each strategy was run 3 times to obtain a converged inference to ensure the accuracy of estimation.

Genetic diversity indices, including the percentage of polymorphic loci (PPL), number of effective alleles [*Ne* = 1/(*p*_2_ + *q*_2_), where *p* = band frequency and *q* = 1 − *p*], expected heterozygosity [*h* = 1 − (*p*_2_ + *q*_2_)], unbiased heterozygosity {*He =* [*N*/(*N*−1)] × *h*, where *N* is sample size}, and Shannon’s information index [*I* = −1 × (*p* × L_n_*p* + *q* × L_n_*q*)], were estimated using the neutral loci by GenAlEx ver. 6.3 [47]. Genetic composition and genetic distinction between the wild populations and cultivated samples of *C. revoluta* and *C. taitungensis* was evaluated by the principle coordinate analysis (PCoA). The Bayesian clustering analysis was also performed to evaluate the degrees of genetic admixture and the genetic structure among populations, using STRUCTURE ver. 2.3.3 [48–50]. The admixture model was used [59]. Posterior probability of the grouping number (*K* = 1~10) was estimated by the Markov chain Monte Carlo (MCMC) method with 10 independent runs to evaluate the consistency of the results, using 3,000,000 steps with a 500,000-step burn-in for each run. The best grouping number was evaluated using Δ*K*[51] by STRUCTURE HARVESTER ver. 0.6.8 [52]. A final 10,000,000 simulations, with a 1,000,000-step burn-in, were executed based on the best *K*.

**Table A1:** Information of sampling sites of *Cycas revoluta* and *C. taitungensis*.

Species	Wild/Cultivated	Adult/Progeny	Sampling Site (Population)	Code	Leaf trait	N	Latitude	Longitude
*Cycas revoluta*	*Wild*	Adult	Iriomote	Iriomote	A	3	24.333708	123.820337
*Cycas revoluta*	*Wild*	Adult	Ishigaki	Ishigaki	A	3	24.337730	124.153348
*Cycas revoluta*	*Wild*	Adult	Yonaguni	Yonaguni	A	3	24.440478	122.985870
*Cycas revoluta*	*Wild*	Adult	Fujian	Fujian	A	3	26.191753	119.537165
*Cycas revoluta*	*Wild*	Adult	Okinawa	Okinawa	A	3	26.207440	127.675227
*Cycas revoluta*	*Wild*	Adult	Amami	Amami	A	3	28.286530	129.385748
*Cycas revoluta*	*Wild*	Adult	Kagoshima	Kagoshima	A	3	31.591265	130.554270

*Cycas revoluta*	Cultivated	Adult	Campus of National Pingtung University of Science and Technology	NPUSTC	A	10	22.638740	120.600127

*Cycas revoluta*	Cultivated	Adult	Notre Dame Heath Farm, Taitung County	StMaC	A	7	22.712072	121.073585

*Cycas revoluta*	Cultivated	Adult	The roadside of Highway No. 11 at 125K	TaiRd11C	A	7	23.012964	121.324124

*Cycas revoluta*	Cultivated	Adult	Taitung Tapo Elementary School	DaPoC	A	5	23.125284	121.230655

*Cycas taitungensis*	Wild	Adult	The preserve area of 40th Compartment of Yen-Ping Area, Taitung County	RL40	B	15	22.857918	120.975018

*Cycas taitungensis*	Wild	Adult	The preserve area of 23rd Compartment of Yen-Ping Area, Taitung County	RL23	B	31	22.867292	121.008930

*Cycas taitungensis*	Wild	Adult	The preserve area of 19th Compartment of Yen-Ping Area, Taitung County	RL19	B	16	22.870879	121.019618

*Cycas taitungensis*	Cultivated	Adult	Mahengheng Blvd., Taitung City	MaHenHenC	B	18	22.771136	121.145511

*Cycas taitungensis*	Cultivated	Adult	Taitung Dulan Elementary School	DuLan01C	B	1	22.877599	121.227522

*Cycas taitungensis*	Cultivated	Adult	A residence house near the Dulan Bridge	DuLan02C	B	1	22.878884	121.230977

*Cycas taitungensis*	Cultivated	Adult	A residence house at Hong-Yeh Village, Taitung County	RL01C	B	4	22.893270	121.066878

*Cycas taitungensis*	Cultivated	Adult	Outside of Taitung Hong-Yeh Elementary School	RL03C	B	13	22.893641	121.063870

*Cycas taitungensis*	Cultivated	Adult	Naruwan Hong-Yeh Hot Spring, Taitung County	RL04C	B	1	22.899838	121.067684

*Cycas taitungensis*	Cultivated	Adult	The intersection of Neighborhoods No. 2 and 3 at at Hong-Yeh Village, Taitung County	RL02C	B	4	22.901864	121.082500

*Cycas taitungensis*	Cultivated	Adult	The office of Longtian Old Folk’s Club, Taitung County	LongTian02C	B	4	22.903850	121.125340

*Cycas taitungensis*	Cultivated	Adult	Taitung Longtian Elementary School	LongTian04C	B	1	22.903850	121.124396

*Cycas taitungensis*	Cultivated	Adult	No.400, Guangrong Rd., Luye Township, Taitung County	LongTian03C	B	1	22.904206	121.126199

*Cycas taitungensis*	Cultivated	Adult	No.23, Shengping Rd., Yanping Township, Taitung County	YenPinC	B	1	22.904280	121.083471

*Cycas taitungensis*	Cultivated	Adult	Longtian Cycad Orchard (private)	LongTian01C	B	12	22.906056	121.123083

*Cycas taitungensis*	Cultivated	Adult	Taitung Lu-Ye Junior High School	LuYeiC	B	2	22.907052	121.135275

*Cycas taitungensis*	Cultivated	Adult	Fuder House at the Yong’an Village, Luye Township, Taitung County	YuanAnn01C	B	1	22.925728	121.124197

*Cycas taitungensis*	Cultivated	Adult	Community Center of Yong’an Village, Taitung County	YuanAnn02C	B	1	22.930631	121.139224

*Cycas taitungensis*	Cultivated	Adult	Taitung Yong’an Elementary School	YuanAnn03C	B	2	22.933397	121.128774

*Cycas taitungensis*	Cultivated	Adult	Ruiyuan Station, Luye Township, Taitung County	JuiYuan02C	B	2	22.953617	121.155438

*Cycas taitungensis*	Cultivated	Adult	Taitung Ruiyuan Elementary School	JuiYuan03C	B	3	22.954660	121.153372

*Cycas taitungensis*	Cultivated	Adult	A residence house at Coastal Range	MtCoastalC	B	4	22.958790	121.183673

*Cycas taitungensis*	Cultivated	Adult	A residence house at Ruiyuan Village	JuiYuan01C	B	5	22.972250	121.164278

*Cycas taitungensis*	Cultivated	Adult	Taitung Tai-yuan junior high school	TaiYuanC	B	4	23.002525	121.289878

*Cycas taitungensis*	Cultivated	Adult	No.45-1, Ganjyulin, Beiyuan, Donghe Township, Taitung County	TongHo01C	B	1	23.004343	121.286874

*Cycas taitungensis*	Cultivated	Adult	Taitung Yuemei Elementary School	YuaMayC	B	1	23.009320	121.148922

*Cycas taitungensis*	Cultivated	Adult	A residence house at Guanshan Township, Taitung County	Guan01C	B	5	23.009578	121.172317

*Cycas taitungensis*	Cultivated	Adult	Visitor Center of the East Coast National Scenic Area Administration	CoastC	B	1	23.025080	121.327515

*Cycas taitungensis*	Cultivated	Adult	Donghe Farm, Taitung County	TongHo02C	B	1	23.037402	121.278763

*Cycas taitungensis*	Cultivated	Adult	Taitung Beiyuan Elementary School	BaiYuanC	B	2	23.040996	121.293182

*Cycas taitungensis*	Cultivated	Adult	Guanshan Junior High School	GuanJrC	B	7	23.044511	121.159973

*Cycas taitungensis*	Cultivated	Adult	Taitung Kanding Elementary School	KanDingC	B	3	23.045083	121.146476

*Cycas taitungensis*	Cultivated	Adult	Guanshan Nursery garden	GuanGDC	B	14	23.046528	121.177194

*Cycas taitungensis*	Cultivated	Adult	Guanshan Station of Farm Irrigation and Engineering Association of Taitung	Guan02C	B	1	23.046861	121.169305

*Cycas taitungensis*	Cultivated	Adult	Guanshan Station of Taitung Forest District Office	Guan03C	B	1	23.047280	121.160681

*Cycas taitungensis*	Cultivated	Adult	Hongshi, , Haiduan Township, Taitung County	RedStone01C	B	1	23.052083	121.169750

*Cycas taitungensis*	Cultivated	Adult	Taitung Hongshi Elementary School	RedStone02C	B	1	23.052083	121.169750

*Cycas taitungensis*	Cultivated	Adult	Taitung Haiduan Elementary School	HaiDuanC	B	3	23.101523	121.176367

*Cycas taitungensis*	Cultivated	Adult	Tung-Fong Nursery Garden (Private)	TongFongC	B	7	23.114431	121.199563

*Cycas taitungensis*	Cultivated	Adult	Taitung Chulai Elementary School	ChuLai02C	B	2	23.117095	121.169028

*Cycas taitungensis*	Cultivated	Adult	Dapu, Chishang Township, Taitung County	ChiShanC	B	4	23.118353	121.213199

*Cycas taitungensis*	Cultivated	Adult	Taitung Farm, Chihshang Township, Taitung County	TaiTungFarmC	B	2	23.119285	121.210892

*Cycas taitungensis*	Cultivated	Adult	Chulai, Haiduan Township, Taitung County	ChuLai01C	B	1	23.124416	121.162591

*Cycas taitungensis*	Cultivated	Adult	Jinping, Haiduan Township, Taitung County	JianPing01C	B	5	23.130533	121.175423

*Cycas taitungensis*	Cultivated	Adult	Taitung Jinping Elementary School	JianPing02C	B	3	23.131481	121.176667

*Cycas taitungensis*	Cultivated	Progeny	Taitung Nursery Garden (Collected by Taitung Forest District Office)	RLF1	B	49	22.775817	121.139128

*Cycas taitungensis*	Cultivated	Progeny	Taitung Nursery Garden (Collected by Guanshan Station of Taitung Forest District Office)	GuanF1	B	16	22.775817	121.139128

*Cycas taitungensis*	Cultivated	Progeny	Hong-Yeh Village Chiefmayor Mr. Hu’s Cycad Orchard	HuF1	B	59	22.975416	121.131056

*Cycas taitungensis*	Cultivated	Progeny	Dianguang Farm at Guanshan Township, Taitung County	DianGuanF1	B	10	22.987972	121.175432

* Leaf trait: A, strongly or moderately keeled leaves and revolute leaflet margins; B, moderately keeled (flatter) leaves and plane leaflet margins.
